# Revision of the *Chaetocnema picipes* species-group (Coleoptera, Chrysomelidae, Galerucinae, Alticini) in China, with descriptions of three new species

**DOI:** 10.3897/zookeys.387.6672

**Published:** 2014-03-10

**Authors:** Yongying Ruan, Alexander S. Konstantinov, Siqin Ge, Xingke Yang

**Affiliations:** 1 Key Laboratory of Zoological Systematics and Evolution, Institute of Zoology, Chinese Academy of Sciences, Beijing 100101, China; 2 Systematic Entomology Laboratory, USDA, ARS, Washington DC, USA; 3 University of Chinese Academy of Sciences, Beijing, 100039, China

**Keywords:** Coleoptera, Alticinae, species group, new species, China, flea beetles

## Abstract

The Chinese *Chaetocnema picipes* species-group is revised. It contains 5 species including 3 new species: *C. cheni* **sp. n.**, *C. constricta* **sp. n.** and *C. kingpinensis* **sp. n.** The lectotype of *C. fortecostata* is designated. A key to all known species of this group from China and the illustrations of habitus and genitalia are provided. A distribution map of species is given.

## Introduction

*Chaetocnema* Stephens, 1831 is a cosmopolitan flea beetle genus with over 400 species known to world (Konstantinov et al. 2011). About 40 species are known to China. Nearctic, Palearctic and Afrotropical faunas of the genus have recently been revised (White 1996, Biondi and D’Alessandro 2006, 2010, Konstantinov et al. 2011), however Chinese *Chaetocnema* species remained mostly unknown.

Two distinct subgenera of *Chaetocnema* are recognized in the Palearctic. They are separated based on the following characters: relative width of the frontal ridge and density and size of punctures on vertex. Since a distinguishing power of these characters weakens significantly in more southern faunas (Biondi 2002, Konstantinov et al. 2011), identification of the subgenera becomes problematic. However, as in many other species rich flea beetle genera (e.g. *Aphthona* Chevrolat), distinct species groups may be recognized in *Chaetocnema*. One of these groups in China is the *picipes* species-group. Five distinct species here attributed to this group share the following characters: 5-7 punctures on vertex close to each eye, two short obscure longitudinal strips without punctures on the base of pronotum, punctures on elytra are arranged in lines, median lobe of aedeagus without deep groove or wrinkles on the ventral surface, apex of aedeagus without obvious denticle, pear-shaped spermatheca.

*Chaetocnema* species of the *picipes* group are usually found in the field feeding on *Rubus*, *Polygonum* and *Solanum*.

We studied all the specimens in IZCAS previously identified as *Chaetocnema concinna* (Marsham) from different provinces of China. It turned out that they are indeed *Chaetocnema picipes*, *Chaetocnema fortecostata* sp. n. or *Chaetocnema constricta* sp. n. *Chaetocnema concinna* is not found in China and all the published records of it in China should be treated as misidentifications.

## Materials and methods

The female genitalia were dissected and mounted onto slides with Hoyer’s medium, photos were taken with digital camera NIKON 5200D attached to the ZEISS AXIOSTAR PLUS Microscope. The photos of habitus were taken with the 5× lens of the same microscope with extra light source softened by semitransparent paper, so as to observe the real color of these tiny beetles. The photos of aedeagus were taken with the KEYENCE VHX-600 microscope. Scanning electron micrographs were taken with FEI QUANTA 450. A map of species distribution was generated by ARCGIS software. Descriptions of species were initially generated by LUCID software, exported from it and extensively edited.

Morphological terminology follows Konstantinov et al. (2011).

Places of distribution of this article are arranged from north to south provinces names in “Materials” paragraphs are in bold font.

Abbreviations: MBL = male body length; MLH = male body length without head; FBL = female body length; FLH = female body length without head; AL/BL = antenna length to body length; MBW = male body width; EL/EW = elytron length (along suture) to width (maximum); PW/PL = Pronotum width (at base) to length; EL/PL = elytron length to pronotum length; EWB/PWB = elytra width at base (in middle of humeral calli) to pronotum width at base; EWM/PWM = maximum width of elytra to maximum width of pronotum.

Abbreviations of collections: BMNH, The Natural History Museum, London, United Kingdom; IZCAS, Institute of Zoology, Chinese Academy of Sciences, Beijing, China; NHRS, Naturhistoriska Riksmuseet, Stockholm, Sweden; USNM, National Museum of Natural History, Washington D.C., USA; ZMAS, Zoological Institute of Russian Academy of Sciences, St. Petersburg, Russia.

## Taxonomy

### *Chaetocnema picipes* species-group

**Diagnosis.** Body small, usually 1.70–2.50 mm. 5–7 punctures on vertex close to each eye. Two short, weakly delineated longitudinal strip without punctures at base of pronotum. All rows of punctures on elytra single and regular, surface between rows smooth and glabrous. Median lobe of aedeagus lacking deep groove or transverse wrinkle on ventral surface, apical dentical weak or absent. Spermatheca pear-shaped or cylindrical, proximal part of spermatheca duct straight. All five species are very similar exteriorly. Color of their bodies and appendages varies between samples collected from different location. The most consistent characteristic to differentiate these five species is the shape of the male genitalia.

Based on the narrow frontal ridge and 5-7 punctures near each eye, species of the *picipes* group can be placed to the *Chaetocnema* subgenus.

#### Key to species of *Chaetocnema picipes* species-group

**Table:** 

1	Body broad. First male protarsomere distinctly larger than second, appendages dark in color, anterolateral angles of pronotum round	2
–	Body narrow. First male protarsomere only slightly larger than second, appendages light in color, anterolateral angles of pronotum obtuse and thickened	4
2	Metatibia proximad to denticle in dorsal view convex, apex of aedeagus subdeltoid, tip of aedeagus broad	*Chaetocnema cheni* sp. n.
–	Metatibia proximad to denticle in dorsal view concave, apex of aedeagus obcordate, tip of aedeagus narrow	3
3	Body bronzish, aedeagus thickened in lateral view	*Chaetocnema fortecostata* Chen, 1939
–	Body copperish, aedeagus narrow in lateral view	*Chaetocnema picipes* Stephens, 1831
4	Body size in male 1.80-2.54 mm and in female 2.11-2.64 mm, length of antenna to length of body about 0.70, pronotum bronzish and elytra blackish brown	*Chaetocnema kingpinensis* sp. n.
–	Body size in male 1.71–1.80 mm and in female 2.15–2.31mm, length of antenna to length of body about 0.62, pronotum and elytra bronzish	*Chaetocnema constricta* sp. n.

#### Chaetocnema (Chaetocnema) picipes

Stephens, 1831

http://species-id.net/wiki/Chaetocnema_picipes

Fig. 1

Chaetocnema picipes Stephens, 1831: 327 (type locality: England, “London” and “Bottisham, Suffolk”; type depository: BMNH; lectotype designated by Booth and Owen 1997: 88).
Chaetocnema chalceola Jacoby, 1885: 731 (type locality: Japan, “Hosokute”; type depository: BMNH; lectotype designated by Konstantinov et al. 2011: 261); Heikertinger 1951: 82, synonymized with *Chaetocnema concinna*.
Chaetocnema laevicollis Thomson, 1866: 229 (type locality: Sweden, “Småland”; type depository: NHRS); Heikertinger 1951: 211, synonymized.
Chaetocnema nitidicollis Jacobson, 1902: 91 (as variety of *Chaetocnema concinna*; type locality: Russia, “Krasnojarsk”; type depository: unknown); Heikertinger 1951: 211, synonymized.
Chaetocnema heikertingeri Lubischev, 1963: 863 (type locality: not given; type depository: ZMAS); Booth and Owen 1997: 88, synonymized.

##### Distribution.

Heilongjiang, Liaoning, Inner Mongolia, Beijing, Hebei, Tianjin, Shanxi, Shandong, Gansu, Qinghai, Shaanxi; Europe, North Asia (Konstantinov et al. 2011); Madgascar (alien) (Biondi 2001).

##### Host plants.

*Polygonum persicaria* Linn. (Polygonaceae), *Polygonum aviculare* Linn., *Brassica rapa* Linn. (Cruciferae) (Fogato and Leonardi 1980); host plant recorded in China: *Polygonum aviculare*.

##### Diagnosis.

*Chaetocnema picipes* very much resembles *Chaetocnema cheni* sp. n. and *Chaetocnema fortecostata* sp. n., but it can be reliably separated from them by the shape of the aedeagus (obcordate on the apex in ventral view and narrow in lateral view) and the copperish color of the body.

##### Description.

MBL = 1.67-1.96 mm; MBH = 1.60-1.80 mm; FBL = 2.01-2.27 mm; MBH = 1.90-2.09 mm; AL/BL = 0.60±0.05; MBW = 1.02–1.13 mm; EL/EW = 2.42–2.49; PW/PL = 1.67–1.68; EWB/PWB = 1.10±0.05; EWM/PWM = 1.40–1.41.

Color of elytra, pronotum and head consistently copperish. Antennomere 1 partly dark brown. Antennomeres 2–3 yellow. Antennomere 4 yellow or partly brown. Antennomere 5 partly brown. Remaining antennomeres black. Pro- and mesofemora brown with yellow on the apex. Metafemora brown. Tarsi brown with yellow on base of each tarsomere.

Base of pronotum with two short, obscure longitudinal impressions without punctures near basal margin. Deep row of large punctures at base of pronotum present on sides, lacking in middle. Pronotal base evenly convex. Lateral sides of pronotum slightly convex with maximum width near base. Anterolateral prothoracic callosity protruding laterally forming round angle. Posterolateral prothoracic callosity projects up to lateral margin of pronotum. Diameter of pronotal punctures 2 to 4 times smaller than distance between them.

Elytra with convex sides. Scutellar row of punctures on elytron regular and single. Remaining rows of punctures regular. Elytral humeral calli well developed. Interspaces between rows of punctures smooth and glabrous. Two lines of minute punctures on each interspace.

Head hypognathous. Frontal ridge between antennal sockets narrow and convex. Frontolateral sulcus present. Suprafrontal sulcus shallow and faint or deep laterally, shallow in middle. Suprafrontal sulcus slightly concave. Orbital sulcus (above the antennal socket) deep, but rather narrow. Width of frontal ridge to width of antennal socket: 0.900–1.005. Width of orbital sulcus to width of frontolateral sulcus: 0.611–0.614. Surface of vertex sparsely and unevenly covered with 6–7﻿﻿﻿ punctures near each eye. Numbers of punctures on each orbit: 2–3. Numbers of setae along frontolateral sulcus on each side: 8–10. Numbers of setae on frons (triangular area surrounded by frontolateral sulci and clypeus): 0. Numbers of setae on clypeus: 7. Numbers of setae on labrum: 6. Anterior margin of labrum slightly concave in middle.

First male protarsomere distinctly larger than second one. First male protarsomere, length to width ratio: 1.63–1.67. First and second male protarsomeres, length to length ratio: 2.00–2.03; width to width ratio: 1.55–1.59. First male protarsomere, width at apex to width at base: 2.58–2.64. Length of metatibia to distance between denticle and metatibial apex: 2.50–2.55. Large lateral denticle on metatibia sharp. Metatibial serration proximal to large lateral denticle present, obtuse. Metatibia proximal to denticle in dorsal view concave. First male metatarsomere, length to width ratio: 3.01–3.05. First and second male metatarsomeres, length to length ratio: 1.87–1.89. First and second male metatarsomeres, width to width ratio about 0.98. Third and fourth male metatarsomeres, length to length ratio: 1.64–1.68. Metatibia length to metafemora length: 0.81±0.05. Length of hind leg to length of body: 0.92±0.05.

Median lobe of aedeagus parallel-sided with apical third slightly widening. Apical part of median lobe in ventral view narrowing abruptly. Ventral longitudinal groove of median lobe absent in apical part and poorly developed in middle and basal part. Apical denticle of aedeagus in ventral view poorly differentiated, straight in lateral view. Minute transverse wrinkles on ventral side of median lobe absent. Median lobe in lateral view narrow and evenly curved. Width (in middle) to length of median lobe (in ventral view) about 0.15.

Spermathecal receptacle pear-shaped. Spermathecal pump much shorter than receptacle. Apex of spermathecal pump cylindrical. Spermathecal pump attached to middle of receptacle top. Maximum width of receptacle situated basally. Basal part of receptacle wider than apical. Posterior sclerotization of tignum spoon-shaped, wider than mid section. Anterior sclerotization of tignum wider than mid section. Apex of vaginal palpus subdeltoid, with lateral side slightly arching. Sides of middle part of vaginal palpus (before apex) narrowing from base, slightly widening towards apex. Anterior sclerotization of vaginal palpus slightly widening anteriorly. Anterior sclerotization of vaginal palpus slightly and evenly curved along length. Anterior end of anterior sclerotization broadly rounded. Length of posterior sclerotization greater than width. Posterior sclerotization about as wide as anterior sclerotization.

**Figure 1.**
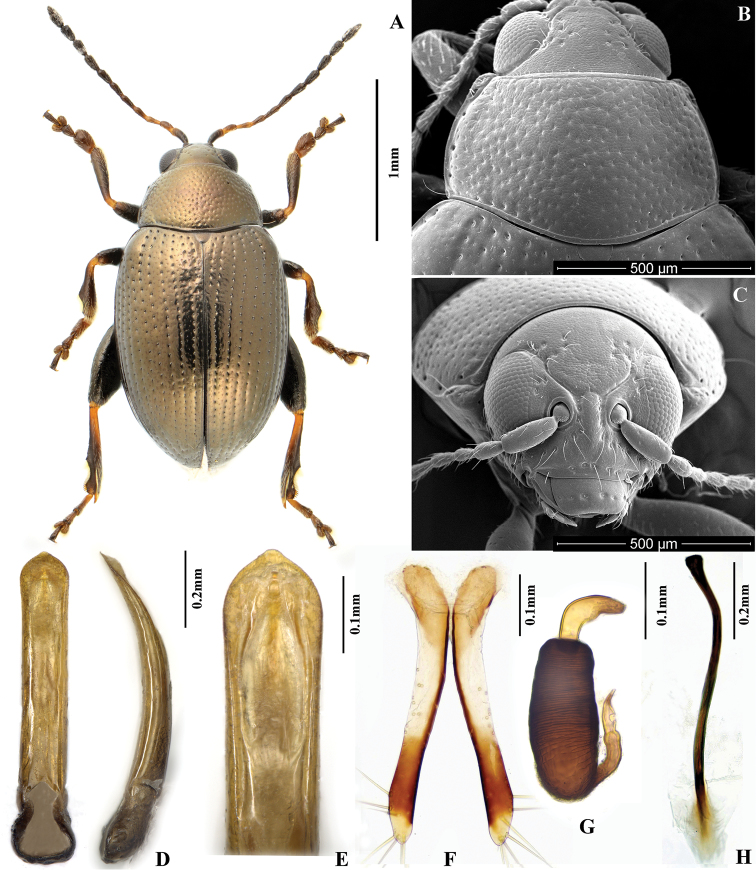
*Chaetocnema picipes*, (Qinling Mountain, Shaanxi, China). **A** Male habitus **B** Pronotum **C** Head **D** Aedeagus, ventral and lateral view **E** Apical part of aedeagus, dorsal view **F** Vaginal palpi **G** Spermatheca **H** Tignum.

##### Materials

**(all the materials preserved in IZCAS):** 1, Harbin, **Heilongjiang**, 11.VI.1965, leg. P. M. Hammond; 2♀1♂, Fujin, **Heilongjiang**, 16.VIII.1970; 15♀3♂, Mishan, **Heilongjiang**, 11-21.VIII.1970; 10♀2♂, Molida, Daxinganling Mountains, **Heilongjiang**, VII-VIII.1970; 1♂, Lingyuan, **Liaoning**; 1♀1♂, Chifeng, **Inner Mongolia**, 8.VIII.1956; 1♂, Fangshan, **Beijing**, leg. Cong; 5, **Beijing**, 5.VII.1980, leg. Subai Liao; 4, **Beijing**, 28.VI.1980, leg. Subai Liao; 14, Zhongguancun, **Beijing**, 8.VI.1962, leg. Shuyong Wang; 15, Yanqing, **Beijing**, 1.VII.1990, leg. Shuyong Wang; 1♀2♂, Shan-hai-Kwan, **Hebei**, 1.IX.1906, leg. F. M. Thomson; 1♀1♂, Xinglong, **Hebei**, 10.VII.1963, leg. Shengqiao Jiang; 1♂, **Tianjin**, 26.IX.1929; 1♀, **Tianjing**, 11.IV.1955; 5♀4♂, **Tianjing**, leg. F. M. Thomson, 1904; 3♀5♂, Lishan National Reserve, **Shanxi**, 112.016°E, 35.420°N, alt.1560m, 26.VII.2012, leg. Yongying Ruan & Zhengzhong Huang, feed on *Polygonum* sp.; 1♀1♂, Long-tong, Tsinanfou (Jinan), **Shandong**; 13♀4♂, Qiujiaba, Wenxian, **Gansu**, alt.2200-2350m, 29.VI.1998, leg. Shuyong Wang; 1♀, Datong, **Qinghai**, V.1956; 13♀28♂, Niubeiliang National Reserve, Qinling Mountain, **Shaanxi**, alt.1690m, 30.VI.2013, leg. Yuanyuan Lu; 4, Niubeiliang National Reserve, Qinling Mountain, **Shaanxi**, alt.1800m, 11.VI.2013, leg.Yongying Ruan; 5♀11♂, Haopingsi National Reserve, Qinling Mountain, **Shaanxi**, 34.095°N, 107.707°E, alt.1200m, 23.VIII.2013, leg. Yongying Ruan; 3♀9♂, Fengxian, Qinling Mountain, **Shaanxi**, 34.2352°N, 106.9572°E, alt.1500m, 21.VIII.2013, leg. Yongying Ruan.

##### Remarks.

This species was recently revised by Booth and Owen (1997) and Konstantinov et al. (2011), and we follow the species status of these two thorough revisions.

We did not find any *Chaetocnema picipes* specimens from South China, it seems that *Chaetocnema picipes* is distributed only in the Palaearctic part of China. The southern boundary of the distribution of *Chaetocnema picipes* is the Qingling Mountain which is also a southern boundary of many other Palaearctic faunistic elements (Yang 2005). We have collected *Chaetocnema picipes* from several places from the north slope of Qinling Mountain during several expeditions, but we did not find any from the south slope. It is also interesting that the specimens collected from Qinling Mountain look darker, the color of the body, appendages, antennomeres and male genitalia are darker than other specimens from other places of northern China.

#### Chaetocnema (Chaetocnema) fortecostata

Chen, 1939

http://species-id.net/wiki/Chaetocnema_fortecostata

Fig. 2

Chaetocnema fortecostata Chen, 1939: 33 (type locality: “Beibei” Guanxi, China; type depository: IZCAS; lectotype designated here); Heikertinger 1951: 207.

##### Distribution.

Shaanxi, Hubei, Chongqing, Sichuan, Zhejiang, Hunan, Jiangxi, Fujian, Yunnan, Guangxi.

##### Host plants.

*Polygonum* sp. (Polygonaceae).

##### Diagnosis.

*Chaetocnema fortecostata* sp. n. is similar to *Chaetocnema picipes* and *Chaetocnema cheni* sp. n. But the aedeagus in lateral view is robust in *Chaetocnema fortecostata* and slender in *Chaetocnema picipes* and *Chaetocnema cheni*. *Chaetocnema fortecostata* have bronzish dorsal surface of its body, while *Chaetocnema picipes* and *Chaetocnema cheni* are copperish.

##### Description.

MBL = 1.75–1.90 mm; MBH = 1.60–1.79 mm; FBL = 2.03–2.12 mm; FBH = 1.89–2.03 mm; AL/BL = 0.64±0.05; MBW = 0.95–1.08 mm; EL/EW = 2.64±0.05; PW/PL =1.62±0.05; EL/PL = 2.98±0.05; EWB/PWB = 1.07–1.19; EWM/PWM = 1.34±0.05.

Color of dorsal side of body bronzish throughout, including head. Antennomere 1 partly dark brown. Antennomeres 2–3 yellow. Antennomere 4 yellow or partly brown. Antennomere 5 partly brown. Remaining antennomeres black. Pro- and mesofemora brown with yellow on apex. Metafemora brown. Tarsi brown with yellow on base of each tarsomere.

Head hypognathous. Frontal ridge between antennal sockets narrow and convex. Frontolateral sulcus present. Suprafrontal sulcus shallow and faint or deep laterally, shallow in middle. Suprafrontal sulcus slightly concave. Orbital sulcus (above antennal socket) obscure and narrow. Width of frontal ridge to width of antennal socket: 0.56-0.66. Width of orbital sulcus to width of frontolateral sulcus: 0.71±0.05. Surface of vertex sparsely and unevenly covered with 5-6 punctures near each eye. Numbers of punctures on orbit: 1-2 on each side. Numbers of setae along frontolateral sulcus: 5-6 on each side. Numbers of setae on frons (triangular area surrounded by frontolateral sulcus and clypeus): 0. Numbers of setae on clypeus: 5. Numbers of setae on labrum: 6. Anterior margin of labrum slightly convex in middle.

Base of pronotum with two short, obscure longitudinal impressions without punctures near basal margin. Deep row of large punctures at base of pronotum present on sides, lacking in middle. Shape of pronotal base evenly convex. Lateral sides of pronotum slightly convex with maximum width near base. Anterolateral prothoracic callosity protruding laterally forming strong round angle. Posterolateral prothoracic callosity projects up to lateral margin of pronotum. Diameter of pronotal punctures 2 to 4 times smaller than distance between them.

Elytra with convex sides. Scutellar row of punctures regular and single. Remaining rows regular. Elytral humeral calli well developed. Interspace smooth and glabrous. 2 lines of minute punctures on each interspace.

First male protarsomere, length to width ratio: 1.65±0.05. First and second male protarsomeres, length to length ratio: 1.94–2.24, width to width ratio: 1.42–1.45. First male protarsomere, width at apex to width at base: 1.60–1.75. Length of metatibia to distance between denticle and metatibial apex: 2.80–2.90. Large lateral denticle on metatibia sharp. Metatibial serration proximal to large lateral denticle present, obtuse. Metatibia proximad to denticle in dorsal view concave. First male metatarsomere, length to width ratio: 3.60–3.67. First and second male metatarsomeres, length to length ratio: 1.87–1.93, width to width ratio: 0.83. Third and fourth male metatarsomeres, length to length ratio: 0.60–0.65. Metatibia length to metafemora length: 0.76±0.05. Length of hind leg to length of body: 0.87±0.05.

Median lobe of aedeagus quite robust and thickened in lateral view. Apical third of median lobe widening evenly. Apical part of median lobe in ventral view narrowing abruptly. Ventral longitudinal groove of median lobe absent in apical part and poorly developed in middle and basal part. Apical denticle of aedeagus in ventral view poorly differentiated, curved ventrally in lateral view. Minute transverse wrinkles on ventral side of median lobe absent. Median lobe in lateral view slightly sinuous near apex. Maximal curvature of median lobe in lateral view situated medially. Width (in middle) to length of median lobe (in ventral view) about: 0.15.

Spermathecal receptacle pear-shaped. Spermathecal pump much shorter than receptacle. Apex of spermathecal pump cylindrical. Spermathecal pump attached to middle of receptacle top. Maximum width of receptacle situated basally. Basal part of receptacle wider than apical. Posterior sclerotization of tignum spoon-shaped, wider than mid section. Anterior sclerotization of tignum narrower than mid section. Apex of vaginal palpus subdeltoid, with lateral side slightly arching. Sides of mid part of vaginal palpus (before apex) narrowing from base, widening towards apex. Anterior sclerotization of vaginal palpus slightly widening anteriorly. Anterior sclerotization of vaginal palpus slightly and evenly curved along length. Anterior end of anterior sclerotization broadly rounded. Length of posterior sclerotization greater than width. Posterior sclerotization about as wide as anterior.

**Figures 2.**
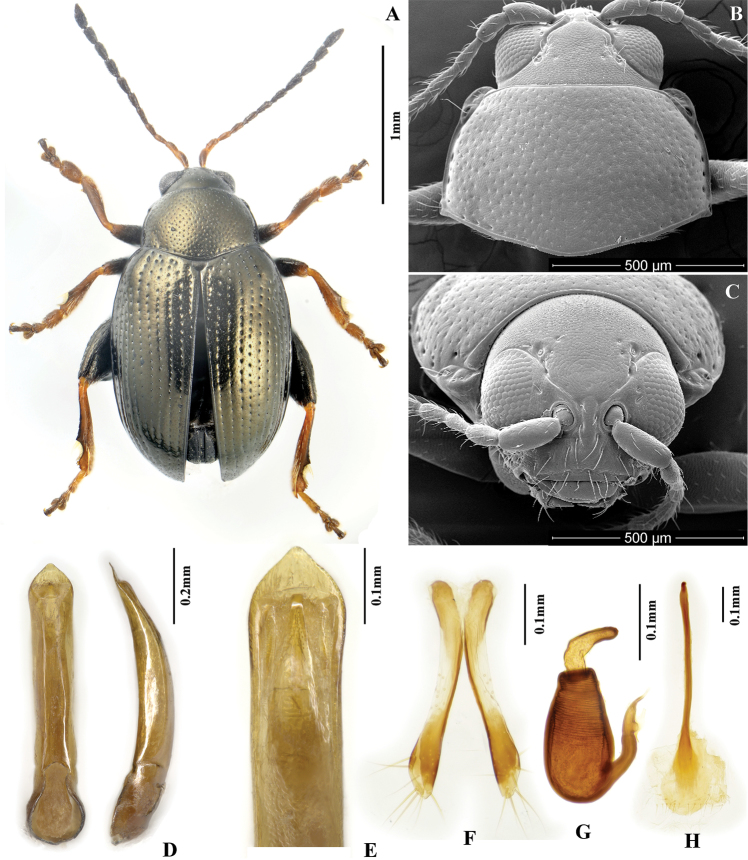
*Chaetocnema fortecostata*. **A** Male habitus **B** Pronotum **C** Head **D** Aedeagus, ventral and lateral view **E** Apical part of aedeagus, dorsal view **F** Vaginal palpi **G** Spermatheca **H** Tignum.

##### Type materials

**(preserved in IZCAS).** Lectotype (designated here): 1♂, (1) Yangshuo, 21.VIII.1938, (2) Lectotype, *Chaetocnema fortecostata* Chen, 1939, des. Yongying Ruan et al.

Paralectotypes (designated here): 2♂5♀, (1) Yangshuo, 21.VIII.1938, (2) Paralectotype, *Chaetocnema fortecostata* Chen, 1939, des. Yongying Ruan et al.

##### Materials

**(all the materials preserved in IZCAS).** 19♀14♂, Huoditang, Qinling Mountain, **Shaanxi**, alt.1600m, 6.VI.2013, leg. Yongying Ruan, feed on *Polygonum* sp.; 3, Maoping, Yangxian, Qinling Mountain, **Shaanxi**, alt.701m, 10.VI.2013, leg. Yongying Ruan; 2♂, Longmen River, Xingshan, **Hubei**, alt.1300m, leg. Shimei Song; 6, Longmen River, Xingshan, **Hubei**, 8.IX.1994, alt.1300m, leg. Jian Yao, feed on *Polygonum* sp.; 1♀2♂, Sanxia Linchang, Badong, **Hubei**, 26.VI.1994, alt.130m, leg. Jian Yao; 2♀1♂, Beibei, **Chongqing**, 17.V.1941; 3♂, Longchi, **Sichuan**, IX.29; 1♀2♂, Fengdu, **Sichuan**, alt.200m, 29.IX.1994, leg. Shimei Song; 56, Wangerbao, Wangxian, **Sichuan**, alt.1200m, 4.X.1994, leg. Jian Yao; 1♀1♂, Chudian, Emei Mountain, **Sichuan**, 28.VI.1957, leg. Fuxing Zhu; 1♀, Tienmo Shan, **Zhejiang**, 20.IX.1953; 24, Shanmuhe, **Hunan**, alt.600, 14.VIII.1988, leg. Shuyong Wang; 7♀4♂, Xingzi, **Jiangxi**, 1932; 1♀1♂, Shuyang, Fuan, **Fujian**, IV.3013, leg. Yongying Ruan, feed on *Polygonum* sp.; 5♀2♂, Sangxiang, Xingcun, Chongan, **Fujian**, alt.740m, 7.VI.1960, leg. Yong Zuo; 187, Baijixun, Weixi, **Yunnan**, alt.1780m, leg. Shuyong Wang, feed on *Polygonum* sp.; 14♀3♂, Xiaomengyang, **Yunnan**, alt.900m, III-IV.1957, leg. Shuyong Wang; 1♂, Damenglong, Xishuangbanna, **Yunnan**, alt.650m, 6.X.1958, leg. Zhizhi Chen.

##### Remarks.

There is no holotype or paratype in the IZCAS. But we found eight specimens belonging to what looks like a type series of this species labeled as “*Chaetocnema fortecostata* sp. n.” with Chen’s handwriting. The locality on the label corresponds with the original description. Therefore we consider these eight specimens as the syntypes. Here we designate one male as the lectotype and the remaining seven as paralectotypes.

This species only occurs in the Oriental China while the northernmost specimens were found on the southern slopes of Qinling Mountain, which is considered as a border between Palearctic and Oriental Regions within China (Chen 1997; Yang 2005).

#### Chaetocnema (Chaetocnema) cheni

Ruan, Konstantinov & Yang sp. n.

http://zoobank.org/3584CDFD-3ACE-4FED-9114-BC746DFEC8B6

http://species-id.net/wiki/Chaetocnema_cheni

Fig. 3

##### Etymology.

We dedicate this species to SH Chen, who originally designated it as new, but left it unpublished. Professor Chen was a classic Chinese entomologist, he laid the foundation for studies of leaf beetles in China.

##### Distribution.

Hunan, Jiangxi, Sichuan, Yunnan.

##### Host plants.

*Solanum tuberosum* Linn. (Solanaceae).

##### Diagnosis.

*Chaetocnema cheni* sp. n. can be differentiated from *Chaetocnema kingpinensis* sp. n. and *Chaetocnema constricta* sp. n. by the following characters: first male protarsomere clearly larger than second, appendages darker in color, anterolateral angles of pronotum round. *Chaetocnema cheni* can be differentiated from *Chaetocnema picipes* and *Chaetocnema fortecostata* based on the following characters: metatibia proximad to denticle in dorsal view convex, apex of aedeagus subdeltoid, tip of aedeagus widely rounded.

##### Description.

MBL = 1.85–2.05 mm; MBH = 1.79–1.93 mm; FBL = 2.10±0.05 mm; FBH = 2.05±0.05 mm; AL/BL = 0.60–0.61; MBW = 1.04–1.06; EL/EW = 1.29; PW/PL =1.47±0.05; EL/PL = 2.91±0.05; EWB/PWB = 1.17±0.05; EWM/PWM = 1.53±0.05.

Color of elytra usually same with or slightly different from pronotum. Color of elytra copperish, sometimes bluish black. Color of pronotum copperish, sometimes bronzish. Head dorsally copperish, sometimes bluish black. Antennomere 1 partly dark brown. Antennomeres 2–3 yellow. Antennomeres 4–5 partly brown. Remaining antennomeres black. Pro- and mesofemora brown with yellow apex. Metafemora brown. Tarsi brown with yellow on base of each tarsomere.

Head hypognathous. Frontal ridge between antennal sockets narrow and convex. Frontolateral sulcus present. Suprafrontal sulcus shallow and faint or deep laterally, shallow in middle. Suprafrontal sulcus slightly concave. Orbital sulcus (above antennal socket) deep. Width of frontal ridge to width of antennal socket: 1.19±0.05. Width of orbital sulcus (above antennal socket) to width of frontolateral sulcus: 0.64–0.67. Surface of vertex sparsely and unevenly covered with 6–7 punctures close to each eye. Numbers of punctures on orbit on each side: 1. Numbers of setae along frontolateral sulcus on each side: 9–10. Numbers of setae on frons (triangular area surrounded by frontolateral sulcus and clypeus): 0. Numbers of setae on clypeus: 7. Numbers of setae on labrum: 6. Anterior margin of labrum slightly concave in middle.

Base of pronotum with two short, obscure longitudinal impressions near basal margin. Longitudinal impressions lack punctures. Deep row of large punctures at base of pronotum present on sides, lacking in middle. Shape of pronotal base evenly convex. Anterolateral prothoracic callosity protruding laterally but poorly developed. Posterolateral prothoracic callosity projects up to lateral margin of pronotum. Diameter of pronotal punctures 2 to 4 times smaller than distance between them.

Elytra with convex sides. All rows of punctures on elytron regular and single. Elytral humeral calli well developed. Interspaces of puncture rows smooth and glabrous. Numbers of minute punctures lines on each interspace: 2.

First male protarsomere distinctly larger than second. First male protarsomere, length to width ratio: 1.50±0.05. First and second male protarsomeres, length to length ratio: 1.69±0.05, width to width ratio: 1.23±0.05. First male protarsomere, width at apex to width at base: 1.87–2.00. Length of metatibia to distance between denticle and metatibial apex: 2.34–2.47. Large lateral denticle on metatibia sharp. Metatibial serration proximal to large lateral denticle present, obtuse. Metatibia proximad to denticle in dorsal view convex. First male metatarsomere, length to width ratio: 2.47–2.68. First and second male metatarsomeres, length to length ratio: 1.58–1.62. First and second male metatarsomeres, width to width ratio: 0.92–1.00. Third and fourth male metatarsomeres, length to length ratio: 0.71±0.05. Metatibia length to the metafemora length: 0.76±0.05.

Median lobe of aedeagus widening gradually towards apex. Apical part of median lobe in ventral view narrowing abruptly forming a subdeltoid apex. Ventral surface of median lobe lateral to median groove apically convex. Ventral longitudinal groove absent in apical and middle part, shallow in basal. Apical denticle of aedeagus in ventral view absent. Apical part of aedeagus in lateral view slightly curved ventrally. Minute transverse wrinkles on ventral side of median lobe absent. Median lobe in lateral view slightly sinusoidal near apex. Median lobe narrow in lateral view. Maximal curvature of median lobe in lateral view situated medially. Width (in middle) to length of median lobe (in ventral view) about: 0.14.

Spermathecal receptacle pear-shaped. Spermathecal pump much shorter than receptacle. Apex of spermathecal pump cylindrical. Spermathecal pump attached to middle of receptacle top. Maximum width of receptacle situated basally. Basal part of receptacle wider than apical. Posterior sclerotization of tignum spoon-shaped, wider than mid section. Anterior sclerotization of tignum wider than mid section. Apex of vaginal palpus subdeltoid, with lateral side slightly arching. Sides of mid part of vaginal palpus (before apex) narrowing from base, slightly widening towards apex. Anterior sclerotization of vaginal palpus slightly widening anteriorly, slightly and evenly curved along length. Anterior end of anterior sclerotization nearly flat. Length of posterior sclerotization greater than width. Width of posterior sclerotization greater to anterior sclerotization.

**Figures 3.**
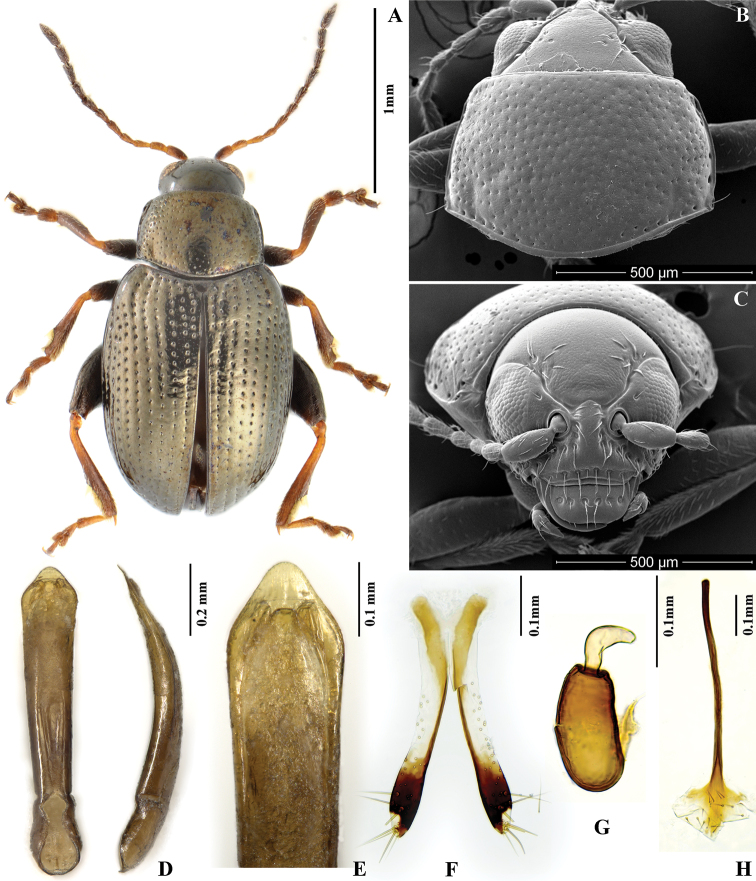
*Chaetocnema cheni*. **A** Holotype, habitus **B** Pronotum **C** Head **D** Aedeagus, ventral and lateral view **E** Apical part of aedeagus, dorsal view **F** Vaginal palpi **G** Spermatheca **H** Tignum.

##### Type materials

**(all the materials preserved in IZCAS**): Holotype: 1♂ (Fig. 3: A), Longling, **Yunnan**, alt.1600m, 1955.V.20, leg. В. Попов (B. Popov). Paratypes: 2♀1♂, Tianping Mountain, Sangzhi, **Hunan**, alt.1370, 1988.VIII.15, leg. Shuyong Wang; 3♀1♂, Jiujiang, **Jiangxi**, 1958.VII-VIII; 2♀, Jiujiang, **Jiangxi**, 1948.VII; 2♀4♂, Jinfou Mountain, **Sichuang**, 1945.VIII.16. leg. Shuyong Wang; 20, Liziping, Wushan, **Sichuan**, alt.1850m, 1993.V.18-19, leg. Youwei Zhang; 4♂5♀, Liziping, Wushan, **Sichuan**, alt.1850m, 1993.VIII.5-6, leg. Xingke Yang, feed on *Solanum tuberosum* Linn.; 1♂, Jinpinghe, **Yunnan**, alt.1700m, 1956V.14, leg. Keren Huang.

##### Remarks.

There is a noticeable variability in body color among studied specimens. The holotype collected from Longling, Yunnan province is copperish in color, but the paratypes from Jinfou Mountain, Sichuang Province have greenish-bronzish pronotum and blue-blackish elytra.

#### Chaetocnema (Chaetocnema) constricta

Ruan, Konstantinov & Yang sp. n.

http://zoobank.org/22CDA31D-F5B5-4207-895A-DCC97EEDE3DF

http://species-id.net/wiki/Chaetocnema_constricta

Fig. 4

##### Etymology.

The name of this species is based on a tiny and tight beetle body.

##### Distribution.

Anhui, Sichuan, Chongqing, Guizhou, Zhejiang, Jiangsu, Jiangxi, Fujian, Yunnan, Guangxi.

##### Host plants.

*Rubus corchorifolius* Linn. f. (Rosaceae), *Rubus fruticosus* Linn., *Polygonum* sp. (Polygonaceae).

##### Diagnosis.

Body of *Chaetocnema constricta* sp. n. usually tiny and narrow. It can be differentiated from *Chaetocnema picipes*, *Chaetocnema fortecostata* sp. n. and *Chaetocnema cheni* sp. n. by the following characters: first male protarsomere only slightly larger than second, appendages light in color, anterolateral angles of pronotum obtuse and thickened. Exteriorly this species resembles *Chaetocnema kingpinensis*. But *Chaetocnema kingpinensis* is larger in body size, having longer appendages and pronotum (relative to body length). If viewed under a soft light, *Chaetocnema constricta*’s body is entirely bronzish, while *Chaetocnema kingpinensis* has usually bronzish pronotum and blackish brown elytra.

##### Description.

MBL = 1.71–1.80 mm; MBH = 1.52–1.65 mm; FBL = 2.15–2.31 mm; FBH = 1.88–2.16 mm; AL/BL = 0.61–0.62; MBW = 0.90–0.94; EL/EW = 1.28±0.05; PW/PL = 1.44±0.05; EL/PL = 2.55±0.05; EWB/PWB = 1.13±0.05; EWM/PWM = 1.37±0.05.

Elytra bronzish, exactly same color as pronotum. Head dorsally bronzish. Antennomere 1 partly dark brown. Antennomeres 2-4 yellow. Antennomeres 5-6 yellow with brown apex. Remaining antennomeres brown with yellow base. Pro- and meso- femora brown with yellow apex. Metafemora brown. Tibia mostly yellow, dark at distal half. Tarsi yellow.

Head hypognathous. Frontal ridge between antennal sockets narrow and convex. Frontolateral sulcus present. Suprafrontal sulcus shallow and faint or deep laterally, shallow in middle. Suprafrontal sulcus slightly concave. Orbital sulcus (above the antennal socket) very deep. Orbital sulcus forming an obvious narrow deep concave above orbit. Width of frontal ridge to width of antennal socket: 0.70–0.75. Width of orbital sulcus (above antennal socket) to width of frontolateral sulcus: 1.20–1.45. Surface of vertex sparsely and unevenly covered with 5–6 punctures on each side close to eye. Numbers of punctures on orbit on each side: 1–2. Numbers of setae along frontolateral sulcus on each side: 9–10. Numbers of setae on frons (triangular area surrounded by frontolateral sulci and clypeus): 0. Numbers of setae on clypeus: 4. Numbers of setae on labrum: 6. Anterior margin of labrum slightly concave in middle.

Base of pronotum with two short longitudinal impressions visible only near basal margin. Longitudinal impressions lack punctures. Deep row of large punctures at base of pronotum present on sides, lacking in middle. Pronotal base evenly convex. Anterolateral prothoracic callosity has finely developed blunt angle protruding antero-laterally. Posterolateral prothoracic callosity projects beyond lateral margin of pronotum. Diameter of pronotal punctures subequal to distance between them.

Elytra with convex sides. Scutellar row of punctures regular and single. Remaining rows regular. Elytral humeral calli well developed. Interspaces of rows of punctures smooth and glabrous. Two lines of minute punctures on each interspace.

First male protarsomere slightly larger than second. First male protarsomere, length to width ratio: 1.90–2.00. First and second male protarsomeres, length to length ratio: 1.60–1.80, width to width ratio: 1.05–1.13. First male protarsomere, width at apex to width at base: 1.70–1.88. Length of metatibia to distance between denticle and metatibial apex: 2.88–3.04. Large lateral denticle on metatibia sharp. Metatibial serration proximal to large lateral denticle present, obtuse. Metatibia proximate to denticle in dorsal view concave. First male metatarsomere, length to width ratio: 1.86–1.91. First and second male metatarsomeres, length to length ratio: 1.91–1.93, width to width ratio: 0.95–1.07. Third and fourth male metatarsomeres, length to length ratio: 0.59–0.76. Metatibia length to metafemora length: 0.82±0.05. Length of hind leg to length of body: 0.91±0.05.

Apical third of median lobe of aedeagus parallel-sided. Apical part of median lobe in ventral view narrowed abruptly and forms big cap on top. Ventral longitudinal groove of median lobe poorly developed, with obtuse margins. Apical part of longitudinal groove as wide as basal. Middle part of longitudinal groove narrower than basal. Apical denticle of aedeagus in ventral view absent. Minute transverse wrinkles on ventral side of median lobe absent. Median lobe in lateral view sinusoidal near apex. Maximal curvature of median lobe in lateral view situated medially. Median lobe thickened in lateral view. Width (in middle) to length of median lobe (ventral view): 0.17.

Spermathecal receptacle pear-shaped and cylindrical. Spermathecal pump much shorter than receptacle. Apex of spermathecal pump cylindrical. Spermathecal pump attached to middle of receptacle top. Basal part of receptacle about as wide as middle and apical parts separately. Posterior sclerotization of tignum spoon-shaped, wider than mid section. Apex of vaginal palpus subdeltoid, with lateral side slightly arching. Sides of mid part of vaginal palpus slightly narrowing from base, and slightly widening towards apex. Anterior sclerotization of vaginal palpus slightly narrowing anteriorly. Anterior end of anterior sclerotization narrowlly rounded. Length of posterior sclerotization greater than width. Posterior sclerotization about as wide as anterior sclerotization.

**Figures 4.**
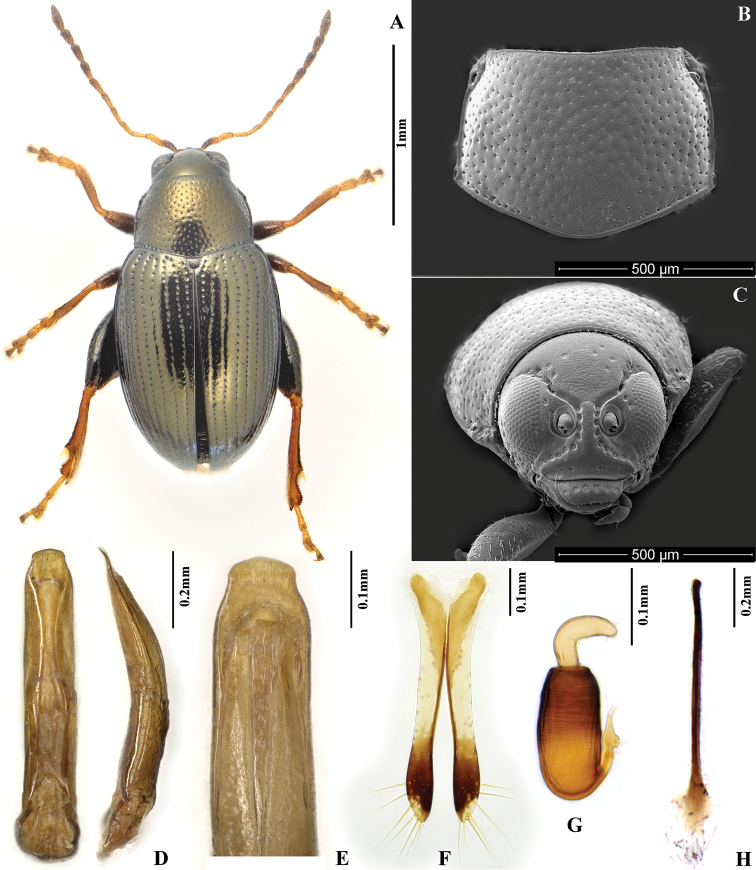
*Chaetocnema constricta*. **A** Holotype, habitus **B** Pronotum **C** Head **D** Aedeagus, ventral and lateral view **E** Apical part of aedeagus, dorsal view **F** Vaginal palpi **G** Spermatheca **H** Tignum.

##### Type materials

**(all the materials preserved in IZCAS).** Holotype: 1♂ (Fig. 4: A), Shuyang, Fuan, **Fujian**, alt.200m, 2013.VIII.12, leg. Yongying Ruan. Paratypes: 30♀20♂, Huangshan, **Anhui**, alt.630m, 18.VIII.1978, leg. Shuyong Wang; 6♀7♂, Shaping, **Sichuan**, 29.XI; 6♀6♂, Ebian, **Sichuan**, X; 1♀, Beibei, **Chongqing**, 11.VI.1940; 6♀3♂, Huaxi **Guizhou**, 8.VI.1980; 6♀1♂, Sanmuping, Tianmu Mountain, **Zhejiang**, 30.VII.1998, leg. Hong Wu; 2♀, Tianmu Mountain, **Zhejiang**, 6.VI.1999, leg. Mingyuan Gao; 3♀1♂, Longwang Mountain, Anji, **Zhejiang**, 1995-1996, leg. Hong Wu; 1♂, Nanjing, **Jiangsu**, 1994, leg. Miao Hu; 20♀8♂, Jiulianshan, **Jiangxi**, 20-23.IX.1978, leg. Peiyu Yu, feed on *Rubus* sp.; 1♂2♀, Dazhulan, **Fujian**, 15-20.VI.1948; 5♀, Wuyi Mountain, Fujian, alt.500-1100m, V.1997, leg. Jiashe Wang; 1♀, Wuyi Mountain, **Fujian**, alt.1200m, 1997.VII, leg. Jiashe Wang; 83♀30♂, Wuyi Mountain, **Fujian**, 5-26.V.1997, leg. Jiashe Wang; 1♀2♂, Nanping, **Fujian**, 22.VII.1957, leg. Jiashe Wang; 1♂, Aotou, Huangkeng, Jianyang, **Fujian**, alt.750–950m, 3.VI.1997, leg.Yong Zuo; 1♀, Longling, **Yunnan**, alt.1600m, 20.V.1995, leg. Zifeng Xue; 1♂, Fangcheng, **Guangxi**, alt.650m, 14.III.1998, leg. Gexia Qiao; 10♀8♂, Jinxiu, **Guangxi**, alt.600m, V.1999, leg. Mingyuan Gao; 5♀2♂, Yanshan, Guilin, **Guangxi**, 15.VI.1963. leg. Shuyong Wang; 1♂, Tianping Mountain, Longsheng, **Guangxi**, 9.VI.1963, leg. Shuyong Wang; 17♀10♂, Yaoshan, Xiuren, **Guangxi**, 6.V.1938.

#### Chaetocnema (Chaetocnema) kingpinensis

Ruan, Konstantinov & Yang sp. n.

http://zoobank.org/2CD2550A-3AE9-4FA8-8658-10E855D21461

http://species-id.net/wiki/Chaetocnema_kingpinensis

Fig. 5

Chaetocnema (Tlanoma) kingpinensis Chen (MS), in Wang 1992: 681.

##### Etymology.

We named this species after a place called “Kingpin” in Yunnan province where some specimens of this species were collected.

##### Distribution.

Jiangxi,Yunnan, Guangxi.

##### Host plants.

*Rubus* sp. (Rosaceae).

##### Diagnosis.

Body of *Chaetocnema kingpinensis* sp. n. quite narrow. It can be differentiated from *Chaetocnema picipes*, *Chaetocnema fortecostata* sp. n. and *Chaetocnema cheni* sp. n. by the following characters: first male protarsomere only slightly larger than second, appendages light in color, anterolateral angles of pronotum obtuse and thickened. This species resembles *Chaetocnema constricta* exteriorly. But *Chaetocnema kingpinensis* is lager in body size, with longer appendages and pronotum (relative to body length). If viewed under a soft light, *Chaetocnema constricta* appears entirely bronze, while *Chaetocnema kingpinensis* usually has bronzish pronotum and blackish brown elytra.

##### Description.

MBL = 1.80–2.54 mm; MBH = 1.66–2.40 mm; FBL = 2.11–2.64 mm; FBH = 1.80–2.45 mm; AL/BL = 0.70; MBW = 0.89–1.04; EL/EW = 1.29–1.29; PW/PL= 1.32; EL/PL = 1.87; EWB/PWB = 1.14; EWM/PWM = 1.45–1.45.

Color of elytra usually differs from color of pronotum. Elytra often brown to black, sometimes bronzish. Pronotum bronzish. Head dorsally dark bronzish. Antennomere 1 yellow but darker than antennomeres 2–5. Antennomeres 2–5 yellow. Antennomeres 6–7 partly brown. Antennomeres 8–11 brown with yellow at base. Tibiae yellow, tasomeres yellow with claw segment brown at apex. Pro- and mesofemora light brown with yellow apex. Metafemora brown.

Head hypognathous. Frontal ridge between antennal sockets narrow and convex. Frontolateral sulcus present. Suprafrontal sulcus shallow and faint or deep laterally, shallow in middle. Suprafrontal sulcus slightly concave. Orbital sulcus (above antennal socket) deep. Width of frontal ridge to width of antennal socket: 0.84–0.88. Width of orbital sulcus (above antennal socket) to width of frontolateral sulcus: 0.93–1.16. Surface of vertex sparsely and unevenly covered with 5–6 punctures close to each eye. Numbers of punctures on orbit on each side: 3–5. Numbers of setae along frontolateral sulcus on each side: 8–10. Numbers of setae on frons (triangular area surrounded by frontolateral sulcus and clypeus): 0. Numbers of setae on clypeus: 7. Numbers of setae on labrum: 6. Anterior margin of labrum slightly concave in middle.

Base of pronotum with two short longitudinal impressions without punctures visible only near basal margin. Deep row of large punctures at base of pronotum present on sides, lacking in middle. Pronotal base evenly convex. Lateral sides of pronotum thickened, only slightly convex with maximum width near base. Pronotum quite convex from lateral view. Anterolateral prothoracic callosity protruding antero-laterally, forms strong obtuse angle. Posterolateral prothoracic callosity projects beyond lateral margin of pronotum. Setae on each callosity long, exceeding half of pronotal length. Wrinkles between punctures on pronotum well developed. Diameter of pronotal punctures subequal to distance between them.

Elytra with convex sides. Scutellar row of punctures regular and single. All other rows of punctures regular. Elytral humeral calli well developed. Interspaces between rows of punctures on elytra smooth and glabrous. Numbers of minute punctures lines on each interspace: 2.

First male protarsomere only slightly larger than second. First male protarsomere, length to width ratio: 1.95–2.03. First and second male protarsomeres, length to length ratio: 1.43–1.52, width to width ratio: 0.89–0.91. First male protarsomere, width at apex to width at base: 1.45–1.55. Length of metatibia to distance between denticle and metatibial apex: 2.73–2.95. Large lateral denticle on metatibia sharp. Metatibial serration proximal to large lateral denticle present, obtuse. Metatibia proximad to denticle in dorsal view concave. First male metatarsomere, length to width ratio: 2.78–2.85. First and second male metatarsomeres, length to length ratio: 1.80–1.90, width to width ratio: 0.92–0.96. Third and fourth male metatarsomeres, length to length ratio: 0.62–0.71. Metatibia length to metafemora length about: 0.89. Length of hind leg to length of body about: 1.04.

Apical third of median lobe of aedeagus parallel-sided. Apical part of median lobe in ventral view narrowing abruptly. Ventral longitudinal groove of median lobe poorly developed in apical and basal part, narrow or absent in middle part. Apical part of longitudinal groove as wide as basal. Apical denticle of aedeagus in ventral view poorly differentiated. Apical denticle of aedeagus in lateral view strongly curved ventrally. Minute transverse wrinkles absent on ventral side of median lobe. Median lobe in lateral view slightly sinusoidal near apex. Maximal curvature of median lobe in lateral view situated medially. Width (in middle) to length of median lobe (in ventral view) about: 0.18. Median lobe narrow in lateral view.

Spermathecal receptacle pear-shaped, slightly narrow in middle. Spermathecal pump much shorter than receptacle. Apex of spermathecal pump cylindrical. Spermathecal pump attached to middle of receptacle top. Maximum width of receptacle situated basally. Basal part of receptacle wider than apical. Posterior sclerotization of tignum spoon-shaped, wider than mid section. Mid section of tignum nearly straight. Anterior sclerotization of tignum wider than mid section. Apex of vaginal palpus subdeltoid, with lateral side slightly arching. Sides of mid part of vaginal palpus (before apex) narrowing from base, slightly widening towards apex. Anterior sclerotization of vaginal palpus slightly narrowing anteriorly. Anterior sclerotization of vaginal palpus slightly and evenly curved along length. Anterior end of anterior sclerotization broadly rounded. Length of posterior sclerotization greater than width. Posterior sclerotization about as wide as anterior sclerotization.

**Figures 5.**
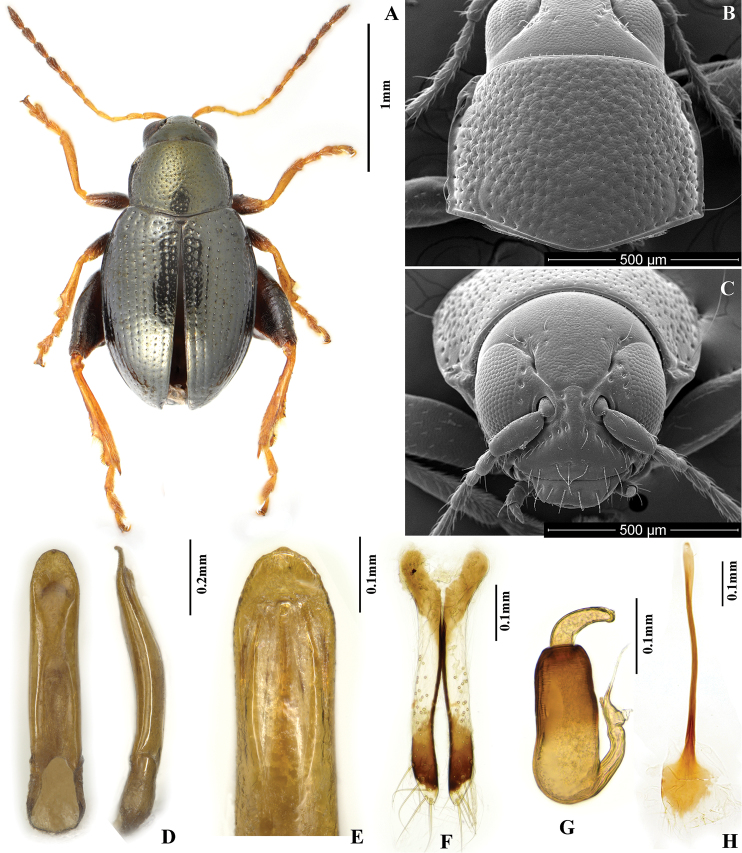
*Chaetocnema kingpinensis*. **A** Holotype, habitus **B** Pronotum **C** Head **D** Aedeagus, ventral and lateral view **E** Apical part of aedeagus, dorsal view **F** Vaginal palpi **G** Spermatheca **H** Tignum.

##### Type materials

**(all the materials preserved in IZCAS).** Holotype: 1♂ (Fig. 5: A), Lushui, **Yunnan**, alt.1900m, 8.VI.1981, leg. Shuyong Wang, feed on *Rubus* sp. 2♀1♂, Jiulianshan national reserve, **Jiangxi**, 8.IX.1978, leg. Youjiao Liu; Paratypes: 16♀11♂, Lushui, **Yunnan**, alt.1900m, 8.VI.1981, leg. Shuyong Wang, feed on *Rubus* sp.; 8♀4♂, Changpotou, Jingping, **Yunnan**, 22.V.1952, leg. Keren Huang et al., feed on *Rubus* sp.; 1♂, Hetouzhai, Jinping, **Yunnan**, alt.2000m, 22.V.1952, leg. Keren Huang et al.; 1♂, Baoshan, **Yunnan**, alt.1600m, 13.V.1955, leg. Bu-xi-ke & Le Wu.; 2♂, Menghun, Menghai, Xishuangbanna, **Yunnan**, alt.1200-1400m, 20-23.V.1958; 1♂, Tiantanshan, Jinxiu, **Guangxi**, alt.600m, 11.V.1999, leg. Mingyuan Gao; 4♀4♂, Tianping Mountain, Longsheng, **Guangxi**, 9.VI.1963, leg. Shuyong Wang; 1♂, Tianping Mountain, Longsheng, **Guangxi**, 740m, feed on *Rubus* sp. 4♀4♂, Tianping Mountain, Longsheng, **Guangxi**, 9.VI.1963, leg. Shuyong Wang; 1♂, Tianping Mountain, Longsheng, **Guangxi**, 740m, feed on *Rubus* sp.

##### Remarks.

This species was originally recognized as new by SH Chen. A series of paratypes were found in the IZCAS collection, but we did not find the holotype. The species was briefly mentioned by Wang (1992). However its name remained unavailable according to the rules of the “International Code of Zoological Nomenclature” (fourth edition). Hence we provide a description for this species keeping the name proposed by Chen.

The specimens of this species collected from Tianping Mountain are extremely large. One male from Guanxi, is 2.54 mm long, and female can be as long as 2.64 mm.

#### Distribution pattern of species of Chinese *Chaetocnema picipes* species-group

Qinling Mountain, considered as a border between Palearctic and Oriental Regions within China (Chen 1997; Yang 2005) seems a natural barrier which separate the Palaearctic species from the Oriental ones. It is also applicable in this species-group. *Chaetocnema picipes* is distributed only in the Palaearctic part of China while *Chaetocnema fortecostata* is distributed only in Oriental China. It is interesting that some of the southernmost specimens of *Chaetocnema picipes* were collected from several places from the north slope of Qinling Mountain but none from the south slope. The northernmost specimens of *Chaetocnema fortecostata* we found are from the southern slopes of Qinling Mountain.

*Chaetocnema cheni* sp. n. seems to be a species in the transition area between Oriental and Palaearctic Region, however *Chaetocnema fortecostata*, *Chaetocnema constricta* and *Chaetocnema kingpinensis* are the Oriental ones.

**Figures 6.**
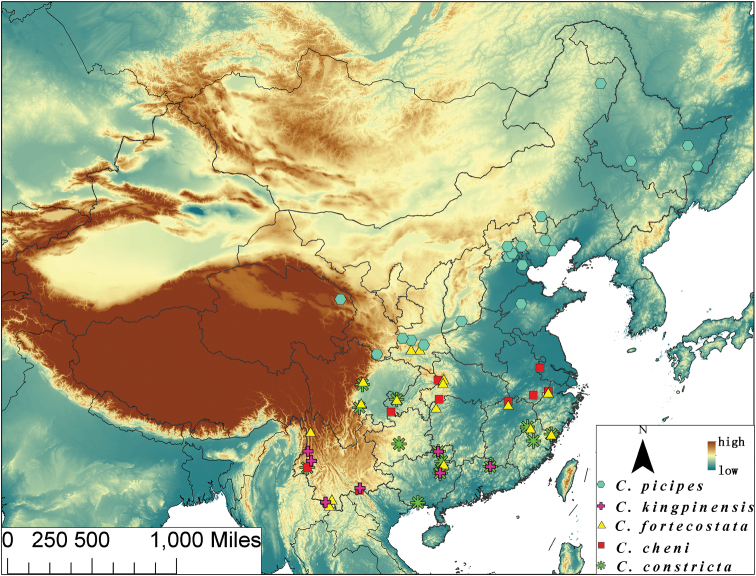
Map of continental China, illustrating localities for distribution of species. *Chaetocnema picipes* = blue hexagons; *Chaetocnema kingpinensis* = purple crosses; *Chaetocnema fortecostata* = yellow triangles; *Chaetocnema cheni* = red squares; *Chaetocnema constricta* = green stars.

## Supplementary Material

XML Treatment for Chaetocnema (Chaetocnema) picipes

XML Treatment for Chaetocnema (Chaetocnema) fortecostata

XML Treatment for Chaetocnema (Chaetocnema) cheni

XML Treatment for Chaetocnema (Chaetocnema) constricta

XML Treatment for Chaetocnema (Chaetocnema) kingpinensis
